# Hypovitaminosis D and its association with lifestyle factors

**DOI:** 10.12669/pjms.315.7196

**Published:** 2015

**Authors:** Mudassar Ali Roomi, Ansa Farooq, Ehsan Ullah, Khalid Parvez Lone

**Affiliations:** 1 Mudassar Ali Roomi, MBBS, M.Phil. Assistant Professor Physiology at Fiji National University, Fiji; 2 Ansa Farooq, M.Sc., M.S. Lecturer Biochemistry at Fiji National University, Fiji; 3 Ehsan Ullah, MBBS, M.Phil. Ph.D. Candidate, Faculty of Health, AUT University, Auckland, New Zealand; 4 Khalid Parvez Lone, Professor and Head Department of Physiology and Cell Biology, UHS, Lahore, Pakistan

**Keywords:** Vitamin D, Physical activity, Sun exposure, Socioeconomicstatus, Indoor jobs, Female gender

## Abstract

**Objectives:**

The present study was designed to determine the serum vitamin D levels and their relation with demographic features and life style factors in young adults.

**Methods:**

It was an analytical cross-sectional study on 88 subjects aged 18–40 years. Relevant information about physical activity, job place, duration of sun exposure, educational status and socioeconomic conditions was obtained. Serum levels of 25-OH vitamin D were measured by ELISA. Data was analyzed using SPSS 20.

**Results:**

Mean serum vitamin D level was 8.44±0.49 (Range: 1.00–21.08) ng/ml in participants. Vitamin D deficiency was found in 98.86% of the population. Mean vitamin D levels were significantly lower in females (*p*=0.0001), physically less active (*p*=0.006), indoor job holders (*p*=0.0001), less sun exposed (*p*=0.001), highly educated (*p*=0.020) and high socioeconomic status (*p*=0.007) bearing and in subjects having relatively fair skin complexion (*p*=0.041).

**Conclusions:**

Serum vitamin D levels of study population were below normal and were associated with female gender, less physical activity, indoor jobs, less sun exposure, higher education and higher socioeconomic class and relatively fair skin complexion.

## INTRODUCTION

Hypovitaminosis D is pandemic.^1^ The occurrence of vitamin D deficiency/insufficiency is high not only in countries where exposure to UV rays is less but as well in countries abutting to the equator because of their traditional and religious clothing habits and life style. Vitamin D supplementation may accordingly be important for improving the health of masses.^2^ It is anticipated that vitamin D deficiency is present in more than one billion people all over the world.^3^ Most of the persons with vitamin D deficiency/insufficiency are symptom free and hence it becomes hard to be detected. Most of the scientists take serum 25(OH)D levels less than 20 ng/ml as cutoff for vitamin D deficiency.^4^

Hypovitaminosis D has been associated with hypertension, cardiovascular diseases (CVD), type-1 and type-2 diabetes mellitus (DM), obesity, dyslipidemia, congestive heart failure, multiple sclerosis, Parkinson disease, rheumatoid arthritis, cognitive impairment, several types of cancers such as prostate, breast and colon cancers, viral and bacterial infections, chronic obstructive pulmonary diseases, autoimmune diseases, dementia and complications of pregnancy.^5^-^7^

Pakistani population is also very deficient in Vitamin D. It is reported that up to 91.50% of some population were found to have hypovitaminosis D in Karachi.^8^ Some other reports also show vitamin D deficiency from various regions of Pakistan. Studies from India report 80–85% prevalence of vitamin D deficiency in local hospital staff and postmenopausal women.^9^,^10^ In a female population of Isfahan, prevalence of vitamin D deficiency was 85%.^11^

The present study was designed to determine the serum vitamin D levels and its relation with demographic features and life style factors in young adults.

## METHODS

This study was conducted from February to December 2012 in the Department of Physiology and Cell Biology, University of Health Sciences, Lahore. It was an analytical cross-sectional study with a sample size of 88 adult subjects aged 18–40 years. Male and female subjects were recruited by convenience sampling method from University of Health Sciences, Lahore, Allama Iqbal Medical College, Lahore, University of the Punjab, Lahore and Amna Inayat Medical College, Sheikhupura. Subjects using calcium and vitamin D supplements, having pregnancy, renal disease, Cushing disease, hypo- and hyperthyroidism, hypo- and hyperparathyroidism and bone problems like osteomalacia and osteoporosis were excluded.

After selection, written informed consent was taken from the subjects. Relevant information i.e. name, age, gender, address etc. was obtained and medical history was taken. Complete general physical and systemic examination was conducted. All the information obtained from the subjects was recorded on the subject data sheet.

Four ml of venous blood was drawn by aseptic technique after 12 hours of overnight fast. Fasting blood sugar (FBS) was measured by glucometer (Gluco Care by General Biological Association). Blood was collected in small vacutainers (yellow top) having gel in it. Blood was centrifuged at 3000 rpm for 10 minutes. Serum was extracted and it was secured in properly labeled 1.5 ml eppendorf tubes for storage at -80°C until assayed. Serum levels of 25-OH vitamin D were measured by an automated EIA analyzer CODA, Bio-Rad laboratories, Hercules, CA, USA using DIA source 25-OH Vitamin D total ELISA Kit (manufactured by DIA source Immuno Assays S.A. Rue du Bosquet, 2, B-1348 Louvain-la-Neuve, Belgium) with Inter assay CV at 26.3 ng/ml is 4.9 % and at 42.0 ng/ml is 4.5%.

Data was analysed with IBM-SPSS version 20. Means of vitamin D levels between the two groups were compared with Independent Sample t-Test. A p-value of ≤0.05 was considered as statistically significant.

### Operational Definitions

### Physical activity

According to the recommendation from the American College of Sports Medicine and the American Heart Association, moderate physical activity is taken as 30 minutes daily activity of moderate severity for at least five days per week. For moderate physical activity, a total of 150 minutes per week aerobic physical activity of moderate intensity is required. There are many ways to get a total of 150 minutes/week. This can be done by performing physical activities in multiple shorter bouts, of at least 10 minutes each, spread throughout the week. Add together the time spent during each of these bouts: e.g. 15 minutes of moderate-intensity activity 10times/week. Such activities include bicycling, brisk walk, water aerobics, playing volleyball, and gardening. Activities less intense than this are taken as mild physical activity and more than this were taken as severe/vigorous physical activity.^12^

### Socioeconomic status (SES)

SES of the study subjects was assessed roughly by the information provided by the subjects. High, middle and low SES was defined on the basis of education, occupation, place of residence and income of the subjects.^13^,^14^

***The skin color*** was measured by Von Luschan’s chromatic scale. This method is used to grade the skin color. In this scale there are 36 boxes ranging from very light to very dark color. We compare the color of these boxes with that of the subject’s skin ideally in a place which is not exposed to the sun (such as under the arm). Most of the Pakistani population has color grades between 15 and 25 on this scale.

## RESULTS

A total of 88 subjects were included in this study of which 38(43%) were females and 50(57%) were males. Their mean serum vitamin D level was 8.44±0.49 (Range: 1.00–21.08) ng/ml and 87 (98.86%) subjects in the study population were found to have hypovitaminosis D.

Males had mean serum vitamin D level 9.98±0.70ng/ml which was significantly higher (P=0.0001) than the mean serum vitamin D level 6.42±0.54ng/ml in females as shown in Table-I. Younger age group (18–25 years old) had mean serum vitamin D level 8.15±0.58 ng/ml whereas other age group (26–40 years old) had mean serum vitamin D level 8.85±0.87 ng/ml. There was no significant difference of means in age groups.

**Table-I:** Association of serum Vitamin D levels with demographic and lifestyle factors.

	Demographic and lifestyle factors	Serum Vitamin D levels (Mean ± SEM)	p value
Gender	Males (n=50)	9.98 ± 0.70	0.0001*
	Female (n=38)	6.42 ± 0.54	
Age	18-25 years (n=50)	8.15 ± 0.58	0.480
	26-40 years (n=37)	8.85 ± 0.87	
Physical activity	Mild activity (n=66)	7.66 ± 0.48	0.006*
	Moderate activity (n=22)	10.76 ± 1.27	
Occupation	Indoor job (n=73)	7.59 ± 0.45	0.0001*
	Outdoor job(n=15)	12.55 ± 1.52	
Socioeconomic status (SES)	Low (n = 14)	11.69 ± 1.66	0.007*
	Middle (n = 68)	8.01 ± 0.50	
	High (n = 6)	5.66 ± 0.76	
Sun exposure	Mild sun exposure (n=60)	7.36 ± 0.51	0.001*
	Moderate sun exposure(n=28)	10.75 ± 0.98	
Consanguineous marriages	Consanguineous parents’ marriage(n=22)	9.88 ± 1.19	0.096
	Non-consanguineous parents’ marriage(n=66)	7.98 ± 0.52	
Education	<Matriculation (n =7)	16.52 ± 1.38	0.020*
	Matric and Intermediate (n =17)	8.98 ± 1.49	
	Graduation and above (n=64)	7.24 ± 0.63	
Milk intake (per day)	Not at all (n= 27)	8.09± 0.81	0.558
	Upto 1 cup /day (n=33)	9.38 ±1.21	
	2 cups / day or more (N=28)	8.29 ± 1.01	
Egg intake (per week)	Not at all (n = 16)	9.77 ± 1.46	0.621
	1 – 5 eggs/week (n = 52)	8.29 ± 0.61	
	> 5 eggs /week (n = 20)	7.80 ± 1.12	
Smoking	Yes (n= 13)	8.58 ± 1.74	0.900
	No (n= 75)	8.41 ± 0.50	

There were significantly lower serum vitamin D levels among physically less active subjects (*p*=0.006), indoor job holders (*p*=0.0001), less sun exposed (*p*=0.001), high socioeconomic status bearing (*p*=0.007) and more educated subjects (*p*=0.020). However, milk intake, egg intake, smoking status and consanguineous parental marriages did not affect the serum vitamin D levels significantly as shown in Table-I.

Most of the subjects had skin complexion grades between 18 and 23 according to von-Luschan’s chromatic scale. There were also significantly lower serum vitamin D levels among subjects having relatively fair skin complexion (*p*=0.041) as shown in Table-II.

**Table-II:** Association between complexion of subjects and serum Vitamin D levels.

von-Luschan’s chromatic scale (two groups)	Frequency (n)	Serum vitamin D (ng/ml) Mean ± SD	p value
15 - 20	48	7.51±4.66	0.041*
21 - 26	40	9.55±4.44	

## DISCUSSION

Serum 25-hydroxy vitamin-D level is considered as the standard to determine hypovitaminosis D and the cut-off level of < 20 ng/ml is most widely used in this regard. We measured serum 25(OH)-D with ELISA technique in an adult, healthy population of Lahore and found that 98.6% subjects had vitamin D deficiency. The frequency of vitamin D deficiency in current study is not astonishing if compared with the previously reported prevalence of hypovitaminosis D from various regions of Pakistan and its neighboring countries.

In a study on female subjects from Karachi, hypovitaminosis D was found in 91.50%.^8^ In another study conducted in mothers and neonates from an urban population of Pakistan vitamin D deficiency was found in 78% mothers and 88% newborns.^15^ A study from rural population of Pakistan reported a median level of vitamin D as 23 ng/ml.^16^ In another study from Pakistan, 84.3% of subjects had low levels of 25-OH vitamin D.^17^ A large population based study on Iranian adult healthy subjects revealed vitamin D deficiency/insufficiency in 79% of the participants.^18^ A report from Qatar presented that 72% of the subjects in their population had vitamin D deficiency.^19^ In studies from Kingdom of Saudi Arabia, vitamin D deficiency/insufficiency is reported in upto 100% of healthy adult population.^20^,^21^

A study from another sunny Middle East country reported that 79.2% of their men and 77.5% of their women had vitamin D deficiency/insufficiency.^22^ All these studies were carried out in sunny countries similar to Pakistan and most of them were conducted on Muslim populations which share common religious and some cultural or traditional dresses; all of them revealed very high prevalence of vitamin D deficiency/insufficiency although some investigators used little different cut-off levels to label vitamin D deficiency. Thus, our finding of 98.6% hypovitaminosis D in current population has enough agreement with previous investigators’ observations. This high prevalence of vitamin D deficiency in the present study may be explained by the fact that 83% of the subjects had indoor jobs and 75% of the subjects had mild physical activities. Moreover, 68% of the subjects had mild sun exposure. Sedentary life style, very less physical activity and lack of sun exposure all contribute to hypovitaminosis D. Lack of food fortification with vitamin D and consumption of traditional diet deficient in vitamin D are amongst the dietary factors that contribute to vitamin D deficiency. In addition to this, none of the subjects included in this study was using vitamin D supplements. Low intake of vitamin D occurs more commonly in countries where diet is deficient in vitamin D and food items are also not fortified. It leads to deficiency/insufficiency of the compound.^23^

In current study, female gender, less physical activity, indoor job, less sun exposure, higher education and high SES were associated with hypovitaminosis D. Whereas age, consanguineous parental marriage, milk intake, egg intake and smoking were not significantly associated with vitamin D deficiency. It has been reported that high SES is a risk factor for hypovitaminosis D.^24^ Karim et al., has concluded that sunlight exposure significantly improves serum vitamin D levels, moreover they also reported that the mean vitamin D levels were significantly different among females residing in downtown and suburbs. High frequency of vitamin D deficiency was observed in females dwelling in downtown.^15^ The prevalence of vitamin D deficiency/insufficiency remained consistent with only little variation when stratified by age.^22^ Traditional and religious clothing habits and life style is also responsible for vitamin D deficiency, particularly in women.^2^

In Pakistan, most of the people, especially females, because of their religious beliefs, wear such a dress that most of the skin is shielded from sunlight, thus preventing the synthesis of vitamin D. This fact has been proven by various other studies from the world. It is obvious that our clothing style, apart from other factors, affects our vitamin D status. Increasing urbanization and industrialization may also reduce exposure to sunlight. Darker skin color in Pakistani population may also hinder proper penetration of UV-B rays required for vitamin D synthesis. All this necessitates to increase dietary intake of vitamin D and to increase exposure to sunlight. An alternate explanation for hypovitaminosis D in Pakistani population may be that a relatively low serum vitamin D level is normal in this population. This may be the unrevealed truth because no serious health problem has been noticed in the studied population that is apparently very low in vitamin D.

In the end, authors support that effective measures to combat the issue of widespread vitamin D deficiency in local population are urgently required as indicated by previous researchers.^8^,^25^

## CONCLUSIONS

Serum vitamin D levels of study population were below normal and were associated with female gender, less physical activity, indoor jobs, less sun exposure, higher socioeconomic status, higher education and relatively fair skin complexion.
